# Supplementary material for: Direct Photochemical Synthesis of Substituted Benzo[b]fluorenes
Source: Org Lett. 2024 Nov 25;26(48):10364–8. doi: 10.1021/acs.orglett.4c03978 (PMC11629382; doi:10.1021/acs.orglett.4c03978)
Supplement: Supplementary file 2 — ol4c03978_si_002.zip [file ol4c03978_si_002.zip › FID for Publication/5q/20240722_RC-2-C-5-131A-F13-14_01/plots/RC-2-C-5-131A-F13-14_FLUORINE_20240722_01_plot01.pdf]

RC-2-C-5-131A-F13-14

Location	16	Pulse sequence	FLUORINE	Temperature	25	Suboperator	RuairiCrawford
Date collected	2024-07-22	Solvent	acetone	Spectrometer	newucd400-vnmrs400	Operator	MBaumann

SAMPLE

date	Jul 22 2024
solvent	acetone
file	/home/data/MBaumann/ucd400/RuairiCrawford/2024/20240722_RC-2-C-5-131A-F13-14_01/RC-2-C-5-131A-F13-14_FLUORINE_20240722_01.fid

ACQUISITION

sw	89285.7
at	1.000
np	178572
fb	47800
bs	2
ss	2
d1	1.000
nt	32
ct	32

TRANSMITTER

tn	F19
sfrq	376.232
tof	9882.0
tpwr	60
pw	5.000

DECOUPLER

dn	C13
dof	0
dm	nnn
decwave	W40_P8012
dpwr	34
dmf	29412

PRESATURATION

satmode	n
wet	n

SPECIAL

temp	25.0
gain	60
spin	0
hst	0.008
pw90	15.000
alfa	10.000

FLAGS

il	n
in	n
dp	y
hs	nn

PROCESSING

lb	0.50
fn	262144

DISPLAY

sp	-76625.3
wp	89285.7
rfl	76625.3
rfp	0
rp	11.0
lp	0

PLOT

wc	251
sc	8
vs	40
th	1

ai cdc ph

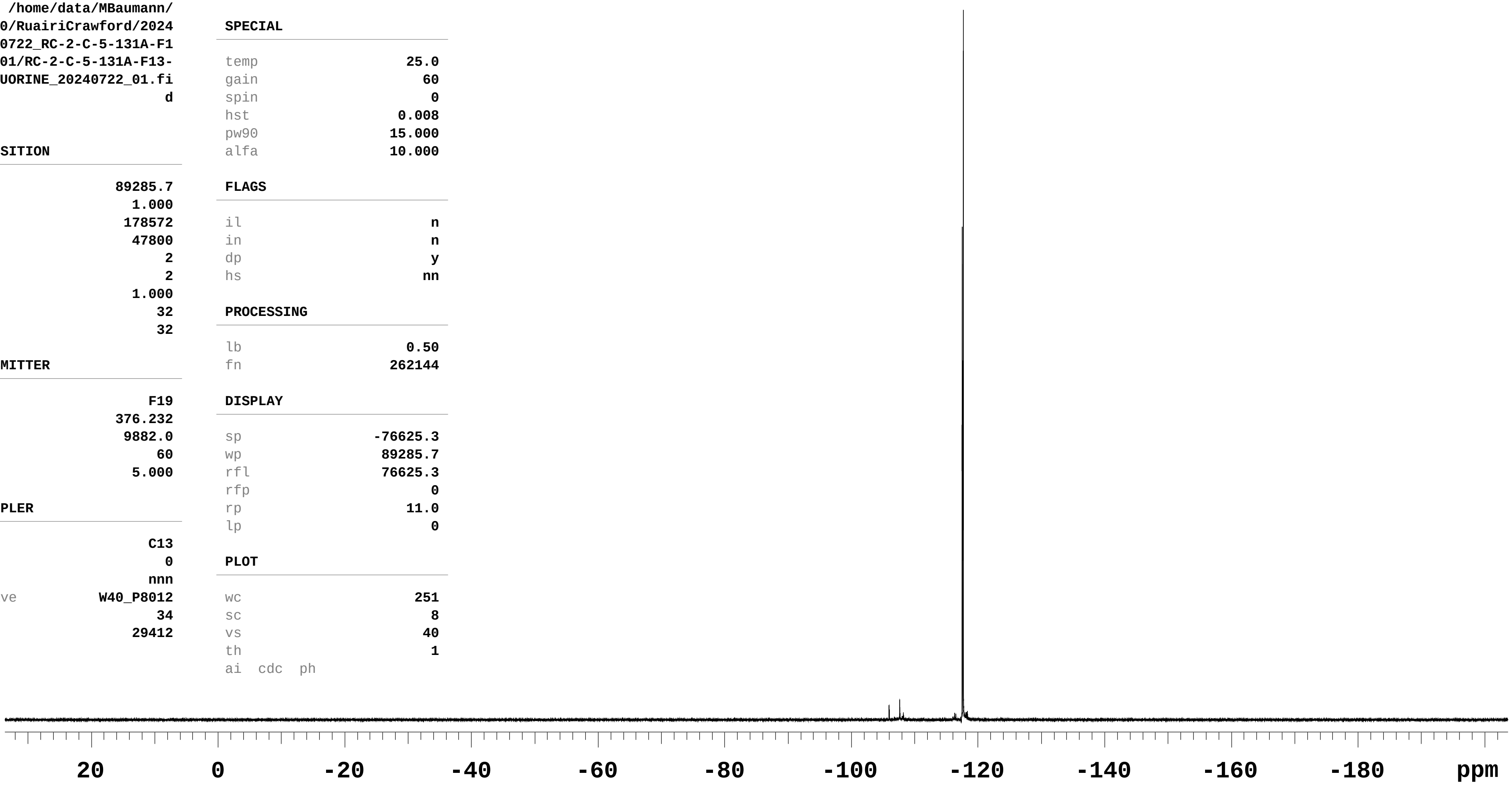

Plotname: RC-2-C-5-131A-F13-14_FLUORINE_20240722_01_plot01

Data file /home/data/MBaumann/ucd400/RuairiCrawford/2024/20240722_RC-2-C-5-131A-F13-14_01/RC-2-C-5-131A-F13-14_FLUORINE_20240722_01.fid

Plot date 2024-07-22
